# Prevalence and Associated Factors of *Parafilaria bovicola* Infection in Cattle in Ebinat District, Northwest Ethiopia

**DOI:** 10.1155/vmi/7641453

**Published:** 2025-10-12

**Authors:** Agabzie Getu, Moges Maru, Nega Yismaw Alemu

**Affiliations:** ^1^Animal Health Department, Ebinat District Livestock and Fisheries Development Office, South Gondar Zone, Ebinat, Amhara Region, Ethiopia; ^2^Department of Veterinary Pathobiology, College of Veterinary Medicine and Animal Sciences, University of Gondar, P.O. Box 196, Gondar, Ethiopia; ^3^Livestock and Fisheries Development Bureau, Bahir Dar, Amhara Regional State, Ethiopia

**Keywords:** associated factors, cattle, Ebinat district, Ethiopia, *Parafilaria bovicola*, prevalence

## Abstract

*Parafilaria bovicola* infection poses a significant health threat to cattle and leads to considerable economic losses due to the condemnation or downgrading of hides. Despite its impact, there is limited information on the prevalence, and associated risk factors regarding the disease in the current study area. A cross-sectional study was conducted from February to December 2024 in Ebinat District, Northwest Ethiopia. The objectives were to estimate the prevalence of *P. bovicola* infection and identify its associated risk factors. A total of 422 cattle were selected using a simple random lottery method. Following clinical examination, exudate samples from the actively bleeding subcutaneous nodules were collected during the early hours of the day. Sedimentation parasitological method, Chi-square(*X*^2^) and univariable logistic regression statistic were used. Overall prevalence of *P. bovicola* infection was found to be 22.04% (93/422; 95% CI: 18.2–26.3). An association between agro-ecology, sex, season, and body condition score and the prevalence of *P. bovicola* infection was shown to be statistically significant (*p* < 0.05). Increased prevalence of the infection with *P. bovicola* was more prevalent in the lowland agro-ecological zone (56.15%) than in the district's midland (6.85%). Infection with *P. bovicola* was significantly more prevalent at the end of the rainy season (31.49%) compared to the dry season (14.94%) (*χ*^2^ = 16.487; *p* < 0.001). Compared to male cattle (14.2%), female cattle had a 2.57-fold increased chance of contracting *P. bovicola* infection (29.9%) (COR = 2.57; 95% CI: 1.58–4.18). In addition, cattle with a poor body condition had a significantly higher prevalence of *P. bovicola* infection (26.8%) compared to those with medium (18.4%) and good (13.8%) body condition (*p* < 0.05). In conclusion, *P. bovicola* infection is prevalent in the study area, particularly in lowland regions. Therefore, location-specific control methods that consider local ecology and seasonal changes should be used.

## 1. Introduction

Bovine parafilariosis, caused by *Parafilaria bovicola*, is a vector-borne filarial nematode infection primarily affecting cattle. Characterized by subcutaneous bleeding nodules commonly known as “bleeding points,” the disease leads to significant economic losses due to hide damage, carcass condemnation, and reduced productivity [1]. The parasite is transmitted by Musca spp. flies (*M. autumnalis* in Europe and *M. vitripennis* in Asia, and *M. xanthomelas*, *M. lusoria* and *M. nevilli* in Africa), which act as intermediate hosts, making its distribution and incidence closely related to climatic and ecological conditions [2–5].

The epidemiology of *P. bovicola* is influenced by climatic conditions favorable to the development and activity of the vector flies. Outbreaks are often seasonal, correlating with warm and humid weather that enhances fly population dynamics [2, 3, 5–7]. Several studies from African countries have reported variable prevalence rates: for example, a prevalence of 15.8% was reported in Nigeria [8], 11.9% in Sudan [9], 20.6% in Kenya [10], 5.6% in central Ethiopia [11], and 20.4% in Northeastern Ethiopia [12].

Risk factors associated with *P. bovicola* infection include age, sex, body condition, husbandry practices, and season and fly control measures [11–13]. The spread of bovine parafilariasis is largely linked to livestock movement from endemic to nonendemic regions [2]. In Europe, the disease became established in Sweden following the importation of infected cattle [14]. It has also been reported in the Netherlands in a bull imported from France [15, 16] and on farms in Belgium [17]. Typical lesions have been observed in Austria, as well as neighboring Germany [18] and Italy [19], with recent re-emergence in 25 of 41 Austrian cattle herds, highlighting the role of vector ecology and animal transportation in disease transmission [20].

In Ethiopia, few studies have investigated the disease, but one study conducted in Raya Kobo reported a prevalence of 20.42% and highlighted associations with a poor body condition, absence of fly control, and communal grazing [12]. Another study in the central highlands found a lower prevalence of 5.6%, suggesting regional variation [11]. Despite these findings, the epidemiology of *P. bovicola* in Ethiopia remains understudied, particularly in areas like Ebinat district, where ecological conditions and livestock management practices may support parasite transmission. Therefore, the objectives of the study were to determine the prevalence of *P. bovicola* infection in cattle and to identify risk factors associated with *P. bovicola* infection in cattle.

## 2. Materials and Methods

### 2.1. Study Area

**Figure 1:**
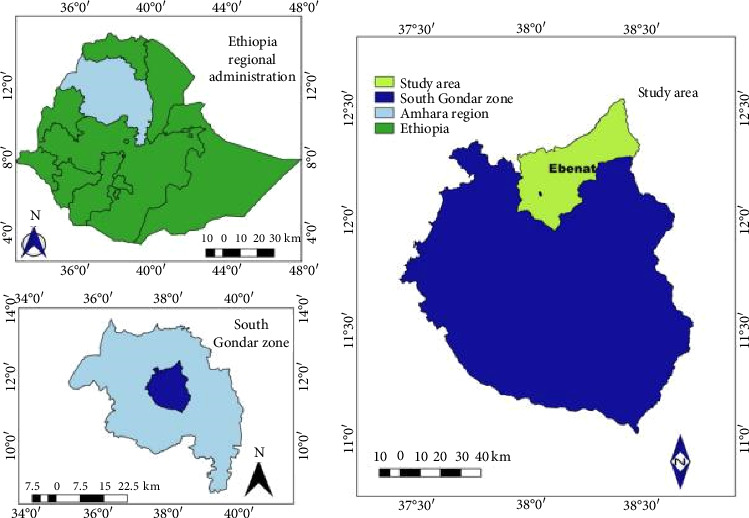
Map of the study area.

The study was conducted in Ebinat district (Figure 1), South Gondar Zone, Amhara region, Northwestern Ethiopia. The area lies at an altitude of 1500–2500 m above sea level (masl), with an average annual rainfall of 1100–1500 mm and mean temperatures ranging from 16°C to 27°C. Ebinat district comprises three agroecological zones: Kolla (35%, 500–1500 masl), characterized by lowland areas with warmer temperatures and lower rainfall; Woyina Dega (50%, 1500–2300 masl), the largest midland zone with moderate and favorable climatic conditions; and Dega (15%, 2300–3300 masl), highland areas with cooler temperatures and higher rainfall. Like most of Ethiopia, Ebinat district has a rainy season from June to August and a dry season from December to February [21]. In the Ebinat district, there are 29 Peasant associations or kebeles and 2 administrative town kebeles. Mixed crop-livestock production is the dominant farming system. The livestock population of the district comprises about 274,327 cattle, 45,040 sheep, 193,019 goats, 834 mules, 105 horses, 28,950 donkeys and 351,487 poultry [22].

### 2.2. Study Population

The study population comprised indigenous and crossbred cattle managed under extensive and semi-intensive production systems. Animals of all age groups and both sexes were included regardless of their body condition or production status. The age of the animals was estimated based on information provided by the owners and dental examination, following the method described by Pace and Wakeman [23]. Body condition was assessed using standard protocols for cattle body condition scoring on a five-point scale, as outlined by Nicholson and Butterworth [24].

### 2.3. Study Design and Sample Size

A cross-sectional study was conducted between February 2024 and December 2024 to investigate the epidemiology of *P. bovicola* infection and identify its associated risk in cattle. The sample size was calculated using the formula provided by Thrusfield [25], assuming a 50% expected prevalence due to the absence of prior data from the study area, with a 95% confidence level and 5% absolute precision:

(1) $$\begin{array}{c} n=\frac{\left(1.96^{2}\times \text{Pexp}\times \left(1-\text{Pexp}\right)\right)}{d^{2}}, \end{array}$$

where *n* is the required sample size, Pexp is the expected prevalence, and *d* is the desired absolute precision.

Therefore, the calculated sample size was 384. To increase precision and account for potential data loss, a **10% buffer** was added, resulting in a final sample size of 422 cattle.

### 2.4. Sampling Technique

The district's lowland and midland agroecological zones were selected purposively based on its high cattle population and preliminary reports from animal health professionals who suspected parafilariasis cases based on clinical signs. Five kebeles at lowland and seven kebeles at midland areas were selected using a simple random sampling technique, following the preparation of a comprehensive list of kebeles. The study animals, cattle, were then selected using a lottery-based simple random sampling method at each kebele.

### 2.5. Data Collection Methods

#### 2.5.1. Clinical Examination

Each animal was examined visually and by palpation for the presence of characteristic bleeding nodules, particularly over the shoulder, neck, and thoracic regions. Cattle with one or more active lesions were considered clinically suspect for *P. bovicola* infection.

#### 2.5.2. Parasitological Diagnosis

Exudates from active lesions were collected aseptically during the early hours of the day when the bleeding is most active. In cases where nodules were present, they were incised using the tip of a sterile scalpel blade to induce bleeding, and blood samples and exudates were collected into test tubes containing 1 mL of 0.85% saline solution. Each sample was properly coded and labeled with relevant animal details, including age category (young or adult), breed (local or exotic), sex, and management system (extensive or semi-intensive). Samples were then transported to the Ebinat Veterinary Clinic laboratory.

Exudate samples were centrifuged for 10 min at 400 rpm in order to concentrate *P. bovicola* free microfilariae or eggs containing microfilariae. Using parasitological diagnostic techniques described by Kaufmann [3] and Urquhart et al. [26], the presence of *P. bovicola* was verified by microscopically detecting free microfilariae, eggs containing microfilariae, or both.

### 2.6. Data Management and Analysis

Microsoft Excel 2007 was used to enter and maintain all of the data gathered for this investigation. The SPSS software (Version 27) was used to analyze the data. The data were presented and summarized using descriptive statistics. Univariable logistic regression analyses were used to evaluate the degree of association between categorical variables and the incidence of *P. bovicola* infection in cattle. Additionally, the association between the seasonal infection rate of *P*. *bovicola* in cattle at both lowland and midland agroecological zones was examined using chi-square (X^2^) test statistics. The results' statistical significance was assessed using a 95% confidence range. If and only if a *p* value of less than 0.05 was regarded as statistically significant.

## 3. Results

### 3.1. Prevalence of *P. bovicola* Infection in Cattle

**Figure 2:**
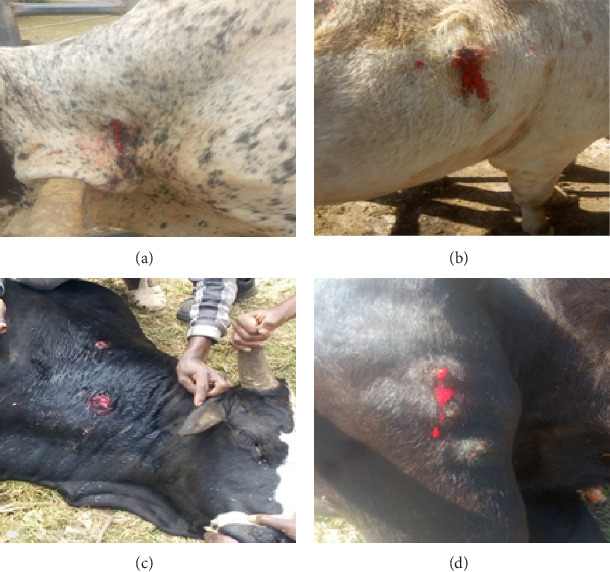
Cutaneous focal hemorrhages on the neck (a, c), thorax (b), and leg (d) of the cattle.

The overall prevalence of *P*. *bovicola* infection in cattle was found to be 22.04% (93/422; 95% CI: 18.2–26.3). Most of the commonly encountered active bleeding spots (focal cutaneous hemorrhages) were observed on the thoracic areas, neck, and legs of cattle infected with *P. bovicola* (Figure 2). During the end of the rainy season (at the end of August and beginning of September), the prevalence of *P. bovicola* infection was higher (31.49%) than it was during the dry season (December, January, and February) (14.94%). There has been a statistically significant variation in prevalence (*X*^2^ = 16.487; *p* < 0.001) (Table 1).

Additionally, during the dry season, the proportional prevalence of *P*. *bovicola* infection in cattle at lowland was 33.69%, which is less than the prevalence that was documented at the end of the rainy season (39.31%) (Table 1). There is statistical significance in this variation (*X*^2^ = 20.252; *p* < 0.05). Furthermore, at midland, the proportional prevalence of *P*. *bovicola* infection in cattle toward the end of the rainy season (7.6%) is lower than the prevalence during the dry season (12.4%) (Table 1). This difference, however, was not statistically significant (*p* > 0.05).

### 3.2. Associated Risk Factors *of P. bovicola* Infection in Cattle

In this study, univariable logistic regression analysis identified agroecology, sex, and body condition score (BCS) as statistically significant risk factors associated with the occurrence of *P*. *bovicola* infection. Age, breed, and management system of cattle were not found statistically significant risk factors for the occurrence of *P. bovicola* infection (*p* > 0.05) (Table 2). The prevalence of *P. bovicola* infection was considerably higher in cattle residing in lowland areas (56.15%) compared to those in midland regions (6.85%). Cattle in lowland zones were found to have a significantly greater likelihood of infection, with an odds ratio of 0.47 (95% CI: 0.29–0.76, *p* = 0.002), indicating that the risk of contracting *P. bovicola* was markedly elevated in lowland areas compared to midland agroecological zones. Female cattle were more affected by *P*. *bovicola* than male cattle, with a prevalence of 29.9% compared to 14.2%, and had a 2.57-fold higher risk of infection (COR = 2.57; 95% CI: 1.58–4.18) throughout the study period (Table 2). In addition, cattle with low body condition scores were 44% more likely to be infected with *P. bovicola* than those with good body scores (COR = 0.44, 95% CI: 0.22–0.88; Table 2).

## 4. Discussion

### 4.1. Overall Prevalence of *P. bovicola* Infection in Cattle

The overall prevalence of *P. bovicola* infection in cattle from the Ebinat district was 22.04% (93/422; 95% CI: 18.2–26.3). This finding aligns closely with reports from Northeastern Ethiopia (20.42%; [12]) and Kenya (20.6%; [10]), suggesting similar ecological conditions, livestock management practices, or vector dynamics across these regions.

However, the prevalence observed in the current study was substantially lower than the rates documented in South Africa, where infection reached 36%–50% in cattle and 34% in buffalo populations within the Greater Kruger National Park complex [27]. Similarly, higher rates were reported in European studies: 35% of young pasture-raised cattle in Sweden exhibited characteristic lesions [28], while PCR-confirmed infections in Austria affected 66.28% (57/86) of sampled lesions from 25 of 41 animal herds [20]. Conversely, the current prevalence finding was markedly higher than the rates reported in other regions, including central highland Ethiopia (5.6%; [11]), Nigeria (15.8%; [8]), Sudan (11.9%; [9]), and Belgium (14.1%; [17]). These disparities may reflect differences in climate, vector prevalence, diagnostic methods (e.g., lesion-based vs. molecular detection), or livestock husbandry practices.

### 4.2. Risk Factors of *P. bovicola* Infection in Cattle

A statistically significant difference in the prevalence of *P. bovicola* infection was identified between cattle in the lowland (56.15%) and midland (6.85%) areas of the Ebinat district (COR = 0.47; 95% CI: 0.29–0.76; *p* < 0.05). This geographic variation aligns with the findings from the Raya Kobo district in northeastern Ethiopia, where *P. bovicola* prevalence was also higher in lowland (22.92%) compared to mid-altitude (17.92%) regions, although the difference was not statistically significant in their report [12]. Similar patterns have been observed in the Transvaal Bushveld of South Africa, seasonal outbreaks of parafilariosis in cattle were consistently recorded in lowland, semi-arid environments, with ovipositional bleeding affecting up to 92.1% of first-year heifers during peak months [29]. The higher prevalence in lowland areas may be attributed to several ecological and management-related factors. Lowland zones often experience warmer temperatures and greater humidity, creating ideal conditions for the survival and reproduction of *Musca* spp. responsible for transmitting the infective larvae of *P. bovicola*. Moreover, cattle in lowland regions are commonly managed under extensive grazing systems, which increase their exposure to vector habitats. This supports evidence from Nigeria that associates lowland ecology—characterized by higher temperatures and humidity—with elevated vector density and greater risk of *P. bovicola* infection [30]. In contrast, Molefe et al. [31] in South Africa found that management practices, such as grazing systems and treatment protocols, played a more influential role in infection dynamics than the ecological zone alone. Additionally, the denser cattle populations and increased mobility in lowland areas—often associated with communal grazing and watering—may facilitate greater vector-host contact and parasite transmission. These factors collectively contribute to the significantly higher infection rates observed in lowland settings.

In the current study, female cattle exhibited a significantly higher prevalence of *P. bovicola* infection (29.9%) compared to male cattle (14.2%) (*p* < 0.05), with the odds of infection in female cattle being 2.57 times higher than that in males (COR = 2.57; 95% CI: 1.58–4.18). This finding aligns with the reports from southern Africa, where higher infection rates in female cattle were attributed to differences in behavior and physiology. Females, particularly during pregnancy or lactation, may have increased exposure due to grazing and more frequent contact with vector-rich environments [29].

Active cutaneous hemorrhages were mainly observed during September to mid-November, coinciding with increased Musca spp fly activity from mid-August to October. During this period, flies were frequently seen feeding on bleeding lesions of infected cattle, supporting their role as biological vectors in the seasonal transmission of *P. bovicola* during the post–rainy season. The study revealed a higher prevalence of *P. bovicola* infection in cattle at the end of the rainy season (31.49%) compared to the dry season (14.94%), with a statistically significant difference (*X*^2^ = 16.487; *p* < 0.001). This seasonal variation aligns with the findings reported in Sudan [32] and Pakistan [33], where increased prevalence was observed during or after the rainy season. Such increases are typically attributed to environmental conditions—such as elevated humidity and moderate temperatures—that enhance vector abundance and activity, particularly among *Musca* spp., which are important in the parasite's transmission. A similar pattern was reported by Ola-Fadunsin et al. [30] in Nigeria, where higher prevalence was associated with wetter months. These findings underscore the role of seasonal climate in shaping transmission dynamics. However, not all studies report this trend. For instance, Sharma et al. [34] in northern India observed no statistically significant seasonal variation, possibly due to consistent climatic conditions or the presence of effective vector control measures, which may mask the seasonal effect.

Regarding the body condition of the cattle, the study found that cattle with a poor body condition had a higher prevalence of *P. bovicola* infection (26.8%) compared to those in good (13.8%) and medium (18.4%) condition having statistically significant variation (*p* < 0.05). This might be due to feeding activity of the filarial nematodes which lead to further weight loss and anemia. By implication, poor body condition exacerbates parasitic infections, while good body condition enhances immunity and resilience [35].

## 5. Conclusion and Recommendations

In conclusion, the study identified a 22.04% prevalence of *P. bovicola* infection among cattle in the Ebinat district, with significant variation based on agroecological zone, sex, season, and body condition. Higher infection rates were notably associated with lowland areas, female cattle, the end of the rainy season, and animals in poor body condition, suggesting that environmental factors and host-related characteristics strongly influence disease distribution. Overall, these results highlight the complex interplay of environmental, seasonal, and management factors that influence the epidemiology of *P. bovicola infection in cattle*. The findings emphasize the need for location-specific control strategies that integrate ecological and seasonal patterns to effectively reduce transmission.

## Figures and Tables

**Table 1:** Association of season and the prevalence of *P*. *bovicola* infection in cattle in Ebinat district.

Season	No.^∗^ of animal examined	No. of positive *n*(%)^∗∗^	*X* ^2^	*p* value
End of rainy	181	57 (31.49)	16.487	< 0.001
Dry	241	36 (14.94)		
Total	422	93 (22.04)		

**Season**	**Infection rate at lowland areas**			
	**No. of animal examined**	**No. of positive n (%)**	**X** ^2^	**p value**

End of rainy	70	52 (39.31)	20.252	< 0.001
Dry	60	21 (33.69)		
Total	130	73 (56.15)		

	**Infection rate at midland areas**			

End of rainy	111	5 (7.6)	1.543	0.214
Dry	181	15 (12.4)		
Total	292	20 (6.85)		

*Note:* No.∗ = number; *n*(%)^∗∗^ = number (percent); *X*^2^ = Chi-square.

**Table 2:** Associated risk factors of *Parafilaria bovicola* infection in cattle at Ebinat district.

Variable	Category	No. of animals	Positive *n* (%)	COR^∗∗^	95% CI	*p* value
Agroecology	Mid	292	20 (6.85)	Ref.		
	Low	130	73 (56.15)	0.47	0.29–0.76	0.002

Age	Young	173	32 (18.5)	Ref.		
	Adult	249	61 (24.5)	1.43	0.88–2.31	0.145

Sex	Male	211	30 (14.2)	Ref.		
	Female	211	63 (29.9)	2.57	1.58–4.18	0.001

Breed	Local	363	76 (20.9)	Ref.		
	Cross	59	17 (28.8)	1.53	0.82–2.83	0.178

BCS	Good	80	11 (13.8)	Ref.		
	Medium	114	21 (18.4)	0.71	0.32–1.56	0.39
	Poor	228	61 (26.8)	0.44	0.22–0.88	0.020

Mgtsyst^∗^	Semi'Insv	43	7 (16.3)	Ref.		
	Extensive	379	86 (22.7)	0.66	0.29–1.54	0.339

*Note:* No. = number; Ref. = reference variable; SemiInsv = semi intensive.

Abbreviation: BCS = body condition score.

^∗^Mgtsyst = management system.

^∗∗^COR = crude odds ratio.

## Data Availability

The datasets used and analyzed during the current study are available from the corresponding author upon reasonable request.
